# Innovative Wound Healing Strategy Through Amorphization of Active Pharmaceutical Ingredients as an Effective Approach for Hydrogel Formulation

**DOI:** 10.3390/ph18101427

**Published:** 2025-09-23

**Authors:** Miłosz Ignacyk, Zbigniew Krasiński, Bozena Michniak-Kohn, Judyta Cielecka-Piontek

**Affiliations:** 1 Department of Pharmacognosy and Biomaterials, Poznan University of Medical Sciences, Rokietnicka 3, 60-806 Poznan, Poland; milosz.ignacyk@student.ump.edu.pl; 2 Department of Vascular, Endovascular Surgery, Angiology and Phlebology, Poznan University of Medical Sciences, 61-701 Poznan, Poland; zkrasinski@ump.edu.pl; 3 Department of Pharmaceutics, Ernest Mario School of Pharmacy, Rutgers, The State University of New Jersey, Piscataway, NJ 08854, USA; michniak@pharmacy.rutgers.edu; 4 Center for Dermal Research, Rutgers, The State University of New Jersey, Piscataway, NJ 08854, USA

**Keywords:** amorphous solid dispersion, hydrogel, dressings, drug delivery, chronic wound, solubility, 3D printing, biological availability

## Abstract

Amorphous solid dispersions (ASDs) provide an effective approach to overcome the poor solubility of many active pharmaceutical ingredients and can facilitate their uniform distribution within hydrogel matrices. Although ASDs are well recognized in oral formulations, their use with hydrogels for wound care remains underexplored. Hydrogels not only offer a biocompatible environment for healing wounds but also are highly versatile for 3D printing, enabling the design of patient-specific dressings customized in composition and structure. This review emphasizes the therapeutic potential of combining ASDs with hydrogel platforms, focusing on how these systems can speed up wound healing, minimize complications, and support personalized therapies. The physicochemical basis for amorphization with limitations and the synergistic effects of bioactive hydrogels are discussed to provide a conceptual basis for advancing this innovative strategy in chronic wound treatment.

## 1. Introduction

Chronic wounds are estimated to affect 1–2% of the population in developed countries, such as the United States, Western Europe, and Australia, with prevalence expected to increase due to aging and rising comorbidities [1,2]. However, a population-based meta-analysis from Europe, Canada, and Australia found a significantly lower prevalence of 0.22%, mainly due to stricter definitions of chronicity and the inclusion of younger, low-risk groups [3]. The rising average life expectancy, along with the increasing proportion of older individuals in modern societies, drives the development of better therapeutic methods for managing chronic wounds. Extended wound healing not only places a medical burden on patients but also creates a significant economic challenge for healthcare systems. According to Medscape, the average cost to treat a single wound is about U.S. $3927, with diabetic foot ulcers being the most expensive, costing nearly twice as much as other chronic wounds. Long-term wound treatments that last approximately two years can cost up to $10,000. Further analysis within the U.S. Medicare system estimated that the total annual expenditure on wound care falls in the range of $28.1 billion and $96.8 billion [4]. In Spain, real-world data from primary care in Barcelona (2015–2017) shows total costs for managing chronic wounds of €35 million, which extrapolates to €1.76 billion annually across the country, with a cumulative prevalence of approximately 0.87% in the population [5]. The pursuit of more effective therapeutic options than those currently available is a natural response to this clinical and economic burden. By potentially increasing the rate of the healing process and reducing the occurrence of complications, such as secondary bacterial infections or amputations, innovative approaches offer a way to alleviate both patient suffering and healthcare costs.

Traditional wound care relies on basic dressing systems, such as gauze and simple moisture-retentive materials, including hydrocolloids, alginates, and hydrofibers. While these dressings provide essential functions, protecting the wound from external contamination, absorbing exudate, and maintaining a moist environment conducive to healing, they often fall short in addressing the more complex requirements of wound management [6,7]. In the context of hard-to-heal wounds, the therapeutic efficacy of these dressings remains limited. Beyond their barrier and absorptive functions, only a few commercially available products contain non-polymeric active ingredients with additional therapeutic properties, typically antiseptics [8,9]. Although some of the dressings, particularly those manufactured from chitosan or gelatin, may exhibit regenerative benefits, these are often insufficient for addressing the multifaceted needs of complex wounds [10,11]. To bridge these therapeutic gaps, the inclusion of pharmacologically active substances is essential.

In this context, it is also valuable to consider natural substances and plant-derived extracts, such as essential oils or polyphenols, that may exhibit activities like antimicrobial, antioxidant, and regenerative properties—highly relevant for wound healing. However, their practical use is often limited by poor solubility and stability [12,13,14,15,16]. Amorphization offers a promising way to overcome these limitations, improving their incorporation into hydrogel-based dressings and expanding their clinical usefulness [17,18,19,20]. This approach aligns with the growing interest in multifunctional natural compounds, whose therapeutic effects often stem not from a single component but from a complex mix of bioactive molecules, generating complementary and multi-directional mechanisms of action [21,22].

Both skin and wounds present challenging environments for drug delivery. The skin’s complex structure and its role as a protective barrier significantly restrict the penetration of most drugs, especially macromolecules and hydrophilic compounds [23]. Conversely, although wounds lack the outermost protective layer of the skin (the stratum corneum), the exposed tissue environment, characterized by hydrophilic interstitial fluid, often hampers drug distribution because many active pharmaceutical ingredients (APIs) have poor aqueous solubility [24]. This solubility barrier, which disqualifies many potentially effective compounds, can be overcome using amorphous solid dispersions (ASDs), a well-established formulation approach in oral drug delivery [25]. When APIs are made more water-soluble through amorphization, they become suitable candidates for incorporation into hydrogel-based dressings, which provide a favorable matrix for wound application [26,27]. This combination offers a dual mechanism of action, enhancing both therapeutic activity and localized delivery.

Enhancing the solubility and permeability of biologically active compounds is not just a technological issue but also has direct clinical significance. The effectiveness of a compound at the target site (e.g., within the skin tissue) depends on reaching local therapeutic concentrations, which is especially difficult for molecules with poor solubility. Similarly, permeability through the skin barrier is more effective when an appropriate concentration gradient is established, allowing the compound to reach this therapeutic level. The benefits of amorphization have been shown by Radeva et al., Yoon et al., and Le et al., who reported not only improved solubility and release of compounds such as curcumin and resveratrol but also in vivo biological effects—such as lower MIC values against pathogens, enhanced fibroblast migration, reduced inflammation, and faster wound healing in animal models, depending on the study [28,29,30]. Although the use of ASDs has mainly been linked to oral drug delivery, recent studies have started to examine their potential in topical and transdermal applications. These new findings show that amorphization can greatly improve the solubility and skin permeability of poorly soluble active ingredients, expanding the therapeutic possibilities of topical formulations [27,31,32,33,34,35,36]. However, their use in wound healing is still not well explored [37,38]. This review focuses on the pharmaceutical concept of amorphous solid dispersions, with a specific focus on their integration into hydrogel matrices as an approach for wound healing. Hydrogels are considered the most suitable carriers for translating this strategy into practice, while 3D printing is presented as an example of how such systems can align with emerging technologies for personalized dressings.

## 2. Wounds—Treatment Approaches and Therapeutic Aims

Skin injuries in individuals without complicating conditions usually heal without the need for additional interventions. However, systemic or local factors can significantly slow down the healing process. Identifying and addressing the root cause is crucial for successful wound closure. It is estimated that nearly 75% of chronic wounds are linked to vascular insufficiency and diabetic foot syndrome [39,40,41,42].

**Figure 1:**
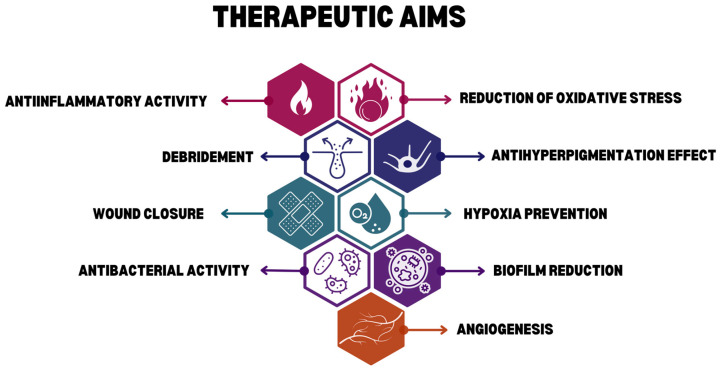
Therapeutic aims for wound healing.

An effective wound management strategy should incorporate a wide range of therapeutic effects that work together to support various stages of healing. These include anti-inflammatory and antioxidant actions that reduce prolonged inflammation and oxidative stress, antibacterial and antibiofilm properties that help control infections and prevent microbial resistance, and debridement support to remove dead tissue and promote tissue rebuilding. Additionally, promoting angiogenesis and improving oxygenation are essential for restoring blood flow and supplying nutrients to healing tissues. Speeding up wound closure helps achieve re-epithelialization quickly and minimizes scarring. The following section provides a detailed explanation of these therapeutic targets (Figure 1), highlighting their physiological significance and impact on the healing process.

Table 1 offers a brief overview of the key pathophysiological mechanisms involved in chronic wounds and their therapeutic importance. It acts as a summary framework that highlights these targets, which can be further explored through amorphization-based strategies. The next chapter provides a more detailed discussion of each mechanism. Readers already familiar with these aspects of pathophysiology may proceed directly to Section 3.

### 2.1. Bacterial Infections

To consider prolonged wounds, we must assume a bacterial contamination has reached a certain level of advancement. While bacteria may not be the initial cause of impaired regeneration, their presence significantly prolongs the healing process [44]. Despite the discomfort from injured tissues, infection introduces additional troubling symptoms such as pain, erythema, swelling, tenderness, and purulent exudate, along with serious systemic effects like fever or sepsis [63,64,65]. A breach of skin integrity is the first step that allows for microbial infection; however, chronic wound pathophysiology further promotes bacterial growth [66]. Necrotic tissue provides a niche for colonization, while local hypoxia favors the proliferation of anaerobic bacteria [67,68]. Furthermore, in many chronic wounds, bacteria persist in biofilms, increasing antibiotic resistance and hindering immune clearance [64]. Additionally, comorbidities such as diabetes or vascular insufficiency impair immune responses and perfusion, further heightening the risk of infection [69,70,71]. Wound regeneration is impossible without eliminating bacteria from the wound site; thus, the importance of this step is paramount [72]. Infection prevention is vital in the primary phase of wound care, where dressings and antiseptics play a key role.

Chronic wounds are predominantly colonized by Gram-negative bacteria, which account for approximately 61% of microbial isolates. Among all identified species, *Staphylococcus aureus* remains the most frequently detected microorganism. A common co-colonizer with *Staphylococcus aureus* is *Pseudomonas aeruginosa*; however, other species, such as *Proteus mirabilis*, *Escherichia coli*, *Acinetobacter baumannii*, and *Klebsiella pneumoniae*, are also frequently found. Among anaerobic bacteria, genera such as *Prevotella*, *Peptoniphilus*, *Peptostreptococcus*, and *Anaerococcus* are commonly isolated [73,74,75]. Moreover, some of the predominant pathogens, particularly *Pseudomonas aeruginosa* and *Acinetobacter baumannii*, are frequently associated with multidrug resistance (MDR), further complicating effective treatment [76,77].

Colonization does not always equal infection; however, bacterial proliferation beyond a critical threshold triggers local and systemic further consequences, such as inflammatory responses [78]. The appearance of infection necessitates a multifaceted approach, given the prevalence of antimicrobial resistance and the often varied nature of infections. Maintaining active concentrations of bactericidal APIs through controlled local release, via hydrogels, nanocarriers, or drug-loaded wound dressings that ensure sustained and site-specific antimicrobial activity, is a significant challenge for successfully managing infected wounds [79].

### 2.2. Biofilm Formation

Bacterial biofilm is a multicellular formation consisting of various bacterial species that adhere to tissue, enclosed in a matrix known as Extracellular Polymeric Substance (EPS). EPS is composed of peptides, polysaccharides, glycolipids, and extracellular DNA [80]. The formation of biofilm is exceptionally organized, particularly in terms of strength, due to chemical communication between the bacterial cells that create this structure. This process is known as quorum sensing and is based on the secretion of autoinducers—chemical signals that help control population density and regulate certain cellular functions [81]. Biofilm formation begins with the adhesion of native bacteria using their natural adhesive structures, such as fimbriae or pili, followed by proliferation that leads to colony germination. The development of colonies is facilitated by EPS production, which helps to form three-dimensional structures. The maturation of microcolonies involves enhanced communication among them, increasing their chances of survival in adverse environments like wound sites affected by immune system cells, ROS, and external antibacterial agents like antibiotics [47]. The biofilm structure serves as a reservoir of bacterial settlers, propagating infection to surrounding tissues and underscoring the urgent need to eradicate this dangerous formation. Unfortunately, this organized form of infection obstructs natural and external antibacterial actions [82]. Immune cells face interrupted access to pathogens, and antibiotics are often ineffective in eradicating them [83]. Furthermore, prolonged inflammation hinders tissue regeneration and can damage the organism’s cells, leading to extended wound healing times. Medical procedures that combat this issue include the removal of necrotic tissue with biofilm using mechanical or enzymatic approaches [47]. Additionally, employing active ingredients against EPS, such as degradable enzymes and quorum sensing inhibitors, provides further support [84]. Advanced dressings that can deliver appropriate debridement with APIs to inhibit further EPS formation offer a chance to improve wound healing [85].

### 2.3. Debridement

Proper wound regeneration involves the natural removal of necrotic cells and non-vital pathogens. It also necessitates replacing dysfunctional tissue components with new cells and extracellular matrix elements. This process promotes healing and is essential for enabling regeneration in chronic wounds [86]. Several methods exist, including invasive surgical and hydrosurgical techniques, larval therapy, and non-invasive approaches such as enzymatic and autolytic solutions [87,88]. A pivotal step involves using dressings that can create the appropriate environment for this process. Furthermore, it may serve as a functional formulation to deliver beneficial active ingredients, such as proteolytic compounds, which can be significantly enhanced by combining two non-invasive debridement techniques [89,90]. Suitable and effective debridement also impacts the microbiological condition, helps keep the wound clear, and strengthens infection healing [91]. The ability to perform debridement effectively at home also facilitates a more supportive therapy environment [92].

### 2.4. Inflammation

Inflammation is a natural response to tissue injuries as well as to microorganisms such as bacteria. This state is essential for physiological healing; however, it can hinder proper recovery when it persists. Cytokines released by platelets and leukocytes trigger a state that enhances the infiltration of immune system cells, including neutrophils, and subsequently monocytes, which differentiate into proinflammatory macrophages (M1). M1 macrophages secrete proinflammatory cytokines—certain interleukins (ILs) such as IL-1, IL-6, IL-12, and tumor necrosis factor α (TNF-α); reactive oxygen species (ROS), and matrix metalloproteinases (MMPs). Non-specific innate immunity is activated to protect the damaged area from infections. Additionally, an inflammatory state creates favorable conditions for the rapid formation of connective tissue and stimulates the migration of fibroblasts and smooth muscle cells. The natural debridement process can occur with the induced presence of immune system cells. Fractured elements can be cleared, and released metalloproteinases may break down the extracellular matrix (ECM) to prepare the wound site for new tissue [69]. Inflammation delineates the area of operation for the immune system and creates favorable conditions to guard against microbial hazards. However, a prolonged inflammatory state can lead to debilitating aftereffects. The overproduction of ROS damages cell membranes and the extracellular matrix [93]. There is an extended discussion on enzymes that degrade the extracellular matrix, impair the composition of tissue elements, and promote further inflammation due to ECM breakdown products. An imbalance between the production and degradation of ECM, along with dysfunctional cell migration and proliferation, results in the entrapment of inflammation in a cycle that is difficult to break without intervention. Despite the benefits for the regeneration process triggered by the inflammation state at the beginning of healing, for prolonged wound regeneration, with surviving inflammation, that state should be reduced [94,95].

### 2.5. Oxidative Stress

Recent findings change our understanding of the significance of oxidative stress in physiological wound regeneration. ROS’ bad reputation cannot eclipse their physiological role in healing skin wounds [96,97]. Neutrophils and macrophages produce them for antibacterial purposes and to prevent the prevalence of infections. Their moderate presence plays a beneficial role in regeneration, helps activate platelets, supports fibroblast proliferation, and promotes proper angiogenesis [98,99,100,101,102]. Nevertheless, the threshold concentration between beneficial roles and harmful effects is fluid and related to the healing phase. Excessive ROS impairs the proliferation and migration of keratinocytes, fibroblasts, and endothelial cells. Platelets’ ability to adhere to the wound site may decrease, influencing the volume of the clot [102,103]. The overproduction of ROS, concerning their damage potential, may also contribute to ECM remodeling and promote fibroblast apoptosis [104,105]. Moreover, the peroxidation of endothelial cell structures leads to reduced angiogenesis at the wound site, resulting in further tissue oxygen debt [106]. Strangely enough, the original purpose of ROS production, when exposed to high levels for prolonged periods, may lead to worse antibacterial activity of immune cells and increase vulnerability to infections [107].

### 2.6. Hypoxia

Moderate hypoxia is a physiological state that serves as a biological cue for wound regeneration, primarily by signaling the need for neovascularization, which is observed through changes in transcription factors such as hypoxia-inducible factor 1 α (HIF-1α) or signal protein Vascular Endothelial Growth Factor (VEGF), angiopoietin-2 (Ang-2), and Stromal Cell-derived Factor-1 (SDF-1) [108,109]. However, prolonged and severe oxygen deficiency is detrimental to proper healing [110]. The origins of this phenomenon are complex and may result from tissue injury that can disrupt microcirculation. Circulatory system insufficiency that can impair blood perfusion, and diseases such as diabetes mellitus or arteriosclerotic disease also play a role [111,112,113]. Additionally, a deficiency may occur due to an increased demand for oxygen in the wound environment, induced by heightened cell activity or bacterial colonization [68,114]. This condition leads to various effects, including decreased cell proliferation in fibroblasts or keratinocytes, reduced ECM remodeling, weakened immune system response, and disrupted redox balance [68,110,115]. Due to the absence of an airtight barrier (lack of stratum corneum), there is an idea to supply the anoxic tissue with oxygen from external sources, such as topical formulations enriched with oxygen (Topical Oxygen Therapy—TOT) or through exposure in a hyperbaric chamber (Hyperbaric Oxygen Therapy—HBOT) [116,117,118]. Furthermore, neovascularization stimuli may be achieved by stabilizing HIF-1α or stimulating the expression of factors like VEGF [108].

### 2.7. Angiogenesis

Proper regeneration of injured tissue is impossible without adequate reconstruction of the blood vessel network. This process is especially vital for wounds with tissue deficits. New vessels deliver nutrients and oxygen to newly created cells and remove metabolites. Disturbances in the vascular system’s function contribute to persistent wound healing issues [119]. Proper angiogenesis involves the activation of endothelial cells, degradation of the basal membrane to enable endothelial cell migration, further formation of capillary structures, and subsequent maturation and stabilization through the recruitment of smooth muscle cells and pericapillary cells [58]. The conditions of prolonged wounds create an environment that hinders normal vessel formation due to the presence of protracted inflammation and excessive expression of angiogenesis inhibitors such as thrombospondin-1 (THBS-1), as well as decreased expression of proangiogenic factors like vascular endothelial growth factor (VEGF) or angiopoietins-1 and -2 (Ang-1, Ang-2) [119]. Ineffective revascularization delays wound regeneration and increases the risk of wound infections [120]. The use of effective dressings and APIs that can promote vascularization in multiple ways is crucial for effective wound management, supporting sustained regeneration.

### 2.8. Wound Closure

Wound closure represents the final phase of wound regeneration, leading to the reconstruction of skin continuity. This process aims to achieve complete reepithelialization and develop epithelial tissue that effectively serves its protective role. First, keratinocytes proliferate, migrate, and differentiate until the wound bed is fully covered. Next, proteins of the dermal–epidermal junction emerge from the edges to the center, establishing the integrity and functionality of the epidermis. Myofibroblasts facilitate wound closure by drawing closer to the surrounding tissue ridges [121]. This situation is typical of secondary closure, which is characteristic of prolonged wounds lacking tissue or exhibiting bacterial infection. The process can be enhanced by external intervention through stitching the wound shortly after injury (primary closure) or indirectly by performing stitching after debridement (delayed primary closure) [122]. Scar covering may be impaired due to factors such as prolonged inflammation or ROS overproduction, which can hinder the proliferation and migration of key cells [54,123]. Moreover, excessive activity of metalloproteinases can obstruct the creation of new ECM and delay the formation of an extracellular scaffold [124]. Additionally, dysfunctional angiogenesis in more complex wounds hampers the effective transport of essential components such as oxygen and nutrients necessary for stable new tissue formation [125,126]. Eliminating dysfunctional factors and furthering natural processes are crucial when the organism is ineffective enough at that final step.

### 2.9. Melanin Overproduction

Pigmentation is a physiological process necessary for its protective features against ultraviolet radiation [127]. The crucial role of melanin production stems from the activity of tyrosinase, the enzyme responsible for melanogenesis from the amino acid tyrosine. Overproduction of pigment occurs when the enzymes involved in melanogenesis, such as tyrosinase-related protein-1 (TRP-1), tyrosinase-related protein-2 (TRP-2), and tyrosinase, are overexpressed [128]. The factors that can stimulate melanogenesis include ultraviolet radiation, oxidative stress, inflammation, certain steroid hormones, and medications, including natural products [129,130,131]. Overproduction of melanin during wound healing is a well-documented phenomenon that affects not only aesthetic effects but also medical reasons. Injury can cause physical discomfort or trauma related to a rough experience. Hyperpigmentation of the scar serves as an additional factor in representing that trauma. Furthermore, the uneven scar in the wound area may impede proper monitoring of skin regeneration [132,133].

## 3. Hydrophobicity: The Domain of Drugs—Amorphization as a Way to Overcome Natural Limitations

### 3.1. From Application Site to Therapeutic Target

Drug delivery is a critical aspect of effective therapy. When considering drug delivery within the skin, it is crucial to understand its complex structure and how this architecture affects molecular permeation. In the case of a wound, the outer physical barriers, especially the stratum corneum, are disrupted. This damage hampers the skin’s primary protective function against external factors and changes the microenvironment of the exposed tissue [134]. In traditional topical drug delivery, formulation design aims to overcome the low permeability of an intact stratum corneum, which favors small, lipophilic molecules. As a result, dosage forms such as ointments, emulsions, and lipogels are often lipophilic themselves to promote diffusion [135]. However, in damaged skin, the pathway for API is different. The application site has altered tissue physiology, and the API faces different transport conditions compared to healthy skin. Temporarily disrupting the skin barrier allows the delivery of less lipophilic molecules or those with limited membrane permeability [136]. Even when transport depends on mechanisms other than passive diffusion, the drug must still reach therapeutically relevant concentrations within the wound area. As the barrier slowly regenerates, achieving and maintaining sufficient solubility at the application site becomes increasingly important because the reformed stratum corneum once again limits diffusion. Therefore, the physicochemical properties of APIs continue to be crucial throughout the healing process, and overcoming solubility limitations remains a significant challenge in pharmaceutical formulation. These challenges can be addressed through amorphization-based approaches, combined with hydrogel systems, which will be discussed in the following section.

### 3.2. Amorphous Solid Dispersions

**Figure 2:**
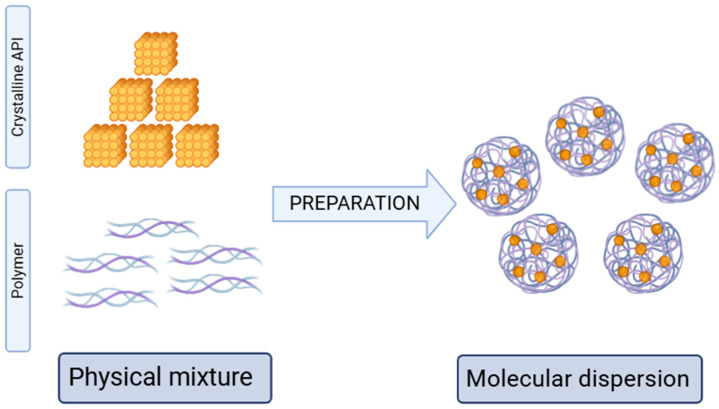
Scheme of preparation of amorphous solid dispersion. Created in BioRender. Karpinski, T. (2025) https://BioRender.com/xwfzkqf.

An amorphous solid dispersion is defined as a dispersion of a drug within an amorphous polymer matrix, where the API is molecularly dispersed (Figure 2). In this context, the API and polymer can be considered as the solute and solvent, respectively, forming a binary system as a homogeneous solid solution with a certain level of thermodynamic stability. The solid-state ASD formulation minimizes the molecular mobility of the API, thereby reducing the likelihood of nucleation and recrystallization [137]. This physical immobilization is crucial for maintaining the amorphous form’s stability over time.

The polymer acts as both a spatial and energetic barrier, separating dispersed API molecules and preventing their aggregation. The primary mechanism by which ASDs inhibit recrystallization relies on molecular interactions between the API and the polymer. Crystal nucleation can be hindered by the entanglement of drug molecules within polymer chains or by disrupting the growth of existing crystal nuclei. A combination of forces, such as hydrogen bonding and van der Waals interactions, creates an energy barrier that must be overcome to initiate recrystallization [138]. The widely accepted concept called the “spring and parachute effect” illustrates this phenomenon. The amorphous API, lacking lattice energy like its crystalline form, dissolves at a much higher concentration. This rapid dissolution is like a stretched spring. The “parachute” represents the stabilizing role of the polymer, which maintains supersaturation by surrounding the dissolved API and preventing its recrystallization. This approach allows the drug to remain above its solubility threshold for longer, enhancing local API availability and therapeutic effectiveness [139]. In addition to improving solubility and achieving localized supersaturation of the incorporated drug, ASDs can also greatly enhance the wettability and dissolution rate of poorly soluble active ingredients [140]. Within the pharmaceutical industry, the ASD concept is well established and has been successfully adopted, as demonstrated by the growing number of market-approved formulations. By 2021, more than 30 ASD-based products had been approved by the U.S. Food and Drug Administration (FDA), highlighting the clinical significance and regulatory acceptance of this approach [141]. Moreover, ASDs are being investigated for use in topical pharmaceutical formulations [142,143,144]. The ongoing development of this technology is further supported by recent research using various amorphization techniques, as outlined in the following section (Table 2).

### 3.3. Preparation Techniques and Technological Advances in ASD Production

Several well-established techniques facilitate the creation of ASDs. The main objective is to preserve the disrupted crystalline structure of the active ingredient in a stabilized format by dispersing it within a polymer matrix, achieved through either solvent evaporation or by cooling a molten mixture of the API and polymer [155]. When using solvents, assessing their safety, regulatory acceptance, and the possibility of residual traces in the final product is critical. From a biological standpoint, water is the most desirable solvent, providing greater safety and compatibility than organic alternatives. While solvent-free methods are an attractive alternative, the elevated temperatures they require may pose a risk to thermolabile APIs [141].

From an industrial perspective, scalability and cost-effectiveness are key factors in selecting an appropriate technique. Spray drying and hot-melt extrusion are commonly used in the pharmaceutical sector due to their efficiency in producing ASDs and their relatively simple implementation [155]. Some techniques offer additional formulation benefits; for example, electrospinning produces nonwoven mats with a high surface area and may enhance wettability [156]. Although supercritical fluid technology produces high-purity, solvent-free products, it is often expensive and difficult to scale up [157,158]. Ultimately, when choosing an amorphization method, consideration should be given to the API’s physicochemical stability, the desired dosage form, and existing manufacturing capabilities. Table 3 summarizes key aspects of selected amorphization techniques that have recently gained increased attention in pharmaceutical research.

## 4. Hydrogels in Wound Therapy

### 4.1. Hydrogels as a Bioactive Matrices

**Figure 3:**
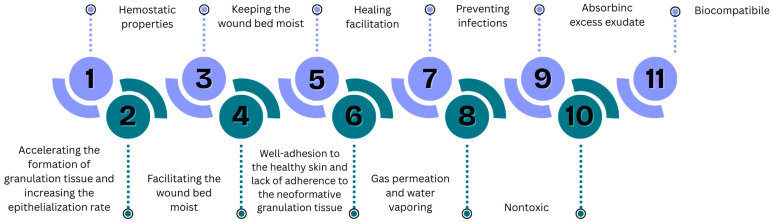
Characteristics of wound dressing [191,192,193,194,195,196,197].

The first consideration of dressings must define the aims we want to reach using a polymer to create a hydrogel form (Figure 3). The innovative approach extends beyond the primary understanding of dressing as physical protection from the external environment.

Hydrogel formulations can be considered as delivery systems for APIs at target sites. They also fulfill critical functions dictated by their structure, such as maintaining a moist wound environment, absorbing excess exudate, and indirectly supporting wound debridement [198]. Synthetic polymers like polyvinylpyrrolidone (PVP) or polyacrylic acid (PAA) are promising candidates for this purpose. Their biological inertness, chemical stability, and ability to form effective gel networks, particularly when cross-linked, make them reliable and practical gelling agents [199,200]. Additionally, their low cost and ease of processing are advantageous for large-scale manufacturing. These polymers can serve as effective matrices for delivering APIs [201]. Notably, some ASD-prepared polymers, such as PVP, also demonstrate hydrogel-forming capabilities, which improve the functionality of hydrogel-based wound dressings and help achieve the therapeutic objectives outlined earlier [202]. Naturally derived polymers capable of forming hydrogels, such as collagen, sodium alginate, or fibroin, often possess inherent regenerative properties that extend beyond moisture retention and exudate absorption [203,204,205,206,207]. However, their use can pose challenges related to physical, chemical, and microbiological stability, as well as contamination risks from their biological sources [208,209,210].

Combined with APIs, these biopolymers can create hybrid systems with broad therapeutic potential, enabling multi-targeted healing actions through complementary mechanisms. Moreover, hybrid hydrogels, comprising blends of synthetic and natural polymers, can be developed when single polymers do not fulfill all formulation requirements. These offer enhanced application properties and provide a flexible platform for wound care formulations. Table 4 summarizes selected synthetic and natural polymers, highlighting their key properties and contributions to wound healing. Below, we characterize representative polymers in terms of properties essential for effective wound healing, which enable the formation of hydrogel dressings.

### 4.2. Polymers

#### 4.2.1. Polyvinylpyrrolidone

Polyvinylpyrrolidone (PVP) is a water-soluble synthetic polymer produced through the radical polymerization of N-vinylpyrrolidone. It is a non-toxic, biocompatible excipient that effectively solubilizes both hydrophilic and lipophilic drugs. Its inert nature and stability across a wide pH range and temperatures make PVP a versatile polymer for pharmaceutical applications. Additionally, PVP can form thin, flexible films on wound surfaces, helping to keep the environment moist, reduce water loss through the skin, and serve as a matrix for controlled release of active ingredients [211]. Beyond its role as an excipient, PVP improves the bioavailability of poorly soluble drugs via methods like hot-melt extrusion (HME) or spray drying, which support the creation and stability of ASDs [242,243]. PVP hydrogels, especially when crosslinked, create three-dimensional networks with enhanced mechanical properties, making them suitable for topical drug delivery in wound healing, especially with additional polymers [244,245]. Although generally non-toxic, allergic reactions have been reported occasionally. Furthermore, as PVP is not biodegradable, it limits its breakdown through natural physiological pathways [246].

#### 4.2.2. Polyvinyl Alcohol

Polyvinyl alcohol (PVA) is a polymer composed of a repetitive structural unit of vinyl alcohol. Like PVP, it is non-toxic, biocompatible, and soluble in water; however, its dissolution requires higher temperature [247]. Its chemical stability, non-immunogenicity, and neutrality make PVA a successful excipient in pharmaceutical formulation. Due to the possibility of creating hydrogels, it can be used to prepare hydrogel dressings to keep the wound bed moist and absorb excess exudate [248]. The possibility of crosslinking PVA enables structural improvements, which are critical to form 3-dimensional structures of dressings; however, when it is not effective enough, mixing with additional polymers enables the obtaining of additional physical properties [249].

#### 4.2.3. Polyacrylic Acid

Polyacrylic acid (PAA) is mainly used as a thickening, suspending, emulsifying, and gelling agent in pharmaceutical formulations [250]. Its ability to form stable, high-viscosity gels at relatively low concentrations offers benefits in developing controlled-release systems. However, its best functional properties are found in crosslinked forms, known as carbomers [251,252]. These crosslinked PAA derivatives are also promising for innovative drug delivery systems, serving as solubilizing agents that enhance the bioavailability of poorly soluble drugs [253]. An additional benefit of PAA is its pH-sensitive swelling property, which can be utilized in designing targeted or site-specific drug delivery systems [254]. Due to its biocompatibility and safety profile, PAA is widely used in topical, oral, and ophthalmic pharmaceutical formulations [255].

#### 4.2.4. Collagen

Collagen is one of the most abundant structural proteins in nature. It can be derived from mammalian sources such as bovine or porcine skin, as well as marine organisms including cuttlefish, octopus, starfish, and algae. However, the use of collagen from mammalian sources is often restricted due to concerns about zoonotic disease transmission, as well as religious or cultural beliefs, and potential immunogenic responses. Though collagen sourced from various origins may exhibit differences in amino acid composition and thermal stability, their overall molecular structures remain comparable [208]. A key distinguishing element includes the risk of transmitting pathogens from animals, such as those causing bovine spongiform encephalopathy (BSE) [208,256]. Collagen is highly processable and can be formulated into films, nanofibers, sponges, or hydrogels. Its complex amino acid structure supports various crosslinking methods that improve the mechanical and physical characteristics of the resulting products, particularly their durability, enabling the creation of 3-dimensional dressings with excellent water retention capabilities [217]. Besides its structural advantages, collagen is naturally biocompatible and biodegradable, making it an excellent choice for wound healing. It fosters cellular adhesion, migration, and proliferation—especially for fibroblasts—thus aiding in tissue regeneration [218]. Its native role in the extracellular matrix ensures smooth integration with surrounding tissue without provoking a significant immune response [214].

#### 4.2.5. Gelatin

Gelatin is a widely used natural polymer obtained through the acidic, basic, or thermal hydrolysis of collagen, mainly type I, sourced from porcine, bovine, or fish skin. It is regarded as a valuable and cost-effective material compatible with human tissues. By mimicking the human extracellular matrix, gelatin offers an appropriate scaffold for the adhesion and migration of keratinocytes and fibroblasts, while demonstrating minimal immunogenicity [257]. Biologically, gelatin is degradable by endogenous gelatinases (MMP-2, MMP-9). It also acts as a template rich in functional amino acids that can be chemically modified into a crosslinked matrix more complex than its original form [258,259]. However, its relatively simple amino acid chain structure makes it susceptible to bacterial degradation. As a less structured derivative of collagen, it has diminished mechanical properties. Despite this, gelatin can be manufactured into various forms, including nanofibers, films, sponges, or gelatin-based polymeric hybrids [220,260]. Significantly, gelatin’s hydrogels are sensitive to heat, which enables the formation of three-dimensional structures that are stabilized by hydrogen bonding when cooled. However, an important limitation is their loss of mechanical strength at body temperature, which could impact the durability of wound healing dressings [261]. Despite its advantages, gelatin exhibits limited long-term structural stability in aqueous environments due to its tendency to swell and dissolve. This requires additional crosslinking or a combination with more stable polymers in hydrogel formulations [221].

#### 4.2.6. Chitosan

Chitosan is a linear polysaccharide made up of β-(1-4)-linked D-glucosamine and N-acetyl-D-glucosamine units, derived from the partial or complete deacetylation of chitin [262]. Chitin is a naturally abundant polymer found in fungi, algae, and the exoskeletons of crustaceans and insects. Due to its limited solubility, chitin requires chemical processing for practical use in biomedical applications. Chitosan has gained attention for its excellent biocompatibility, biodegradability, low immunogenicity, and nontoxic nature, along with its ease of availability and processability [263]. In wound dressings, chitosan plays a multifaceted role in supporting tissue regeneration. It promotes blood clotting by stimulating erythrocyte and platelet aggregation and inhibiting fibrinolysis, thereby prolonging the hemostatic phase. It aids in bacterial clearance from the wound site during the inflammatory phase. In the later stages of healing, chitosan supports granulation tissue formation and promotes re-epithelialization [224]. Unlike amino acid-based polymers like gelatin, chitosan is resistant to bacterial degradation and has inherent antibacterial properties. These antimicrobial mechanisms include disrupting bacterial membranes, chelation of essential metal ions, interfering with DNA replication, and forming a protective film around bacterial cells. Furthermore, chitosan modulates local immune responses by suppressing the production of pro-inflammatory cytokines, including TNF-α [225,264]. Its chemical structure allows for a wide range of modifications, including crosslinking or the conjugation of functional groups, which can enhance its mechanical strength, bioactivity, and stability under physiological conditions [262].

#### 4.2.7. Sodium Alginate

Sodium alginate is a hydrophilic, biocompatible, and biodegradable polymer obtained from brown algae. It exhibits excellent gel-forming capabilities, which are crucial for creating effective wound-healing environments. The polymer consists of blocks of (1,4)-linked β-D-mannuronic acid (M) and α-L-guluronic acid (G) residues. These blocks can consist of consecutive G residues, consecutive M residues, or alternating sequences of G and M, a structural aspect that affects the polymer’s physical properties [265]. One of the most remarkable features of sodium alginate is its capacity to form hydrogels through ionotropic gelation, where mostly divalent cations like Ca^2+^ facilitate cross-linking and network development [266]. The swelling behavior of alginate-based hydrogels varies according to the concentrations of both the polymer and cross-linking ions, enabling the customization of hydrogel properties to address specific wound care needs [230]. Mechanically, additional chemical modifications can enhance properties such as elasticity and tensile strength, improving alginate dressings’ durability and functionality [267]. Furthermore, alginate matrices can be incorporated with bioactive compounds, adding functionalities like anti-inflammatory effects or controlled drug release—features especially beneficial in chronic wound treatment [268]. When ions are used for cross-linking, the resulting hydrogels may gradually degrade through ion exchange with wound exudate (for instance, Na^+^), potentially impacting the dressing’s integrity and the wound healing process [269]. Due to its biocompatibility, moisture retention capacity, antioxidant properties, and versatility in injectable or moldable forms, sodium alginate serves as a highly adaptable polymer for creating advanced wound care solutions [231,270].

#### 4.2.8. Silk Fibroin

Silk fibroin is a structural protein produced by various arthropods; however, the most significant sources for biomedical applications are silkworms (Bombyx mori) and spiders [271,272]. Natural silk fibers are coated with sericin, an amorphous polypeptide that acts as a glue, which is typically removed through degumming to obtain pure fibroin [273]. A key feature of fibroin is its excellent biocompatibility, ensuring safe interactions with living tissues and minimal immune response. Many studies confirm its ability to support cell adhesion, growth, and function, especially when processed into forms like films, scaffolds, hydrogels, sponges, or nanofibers [234,235,236]. Advanced techniques also allow the production of microspheres and nanoparticles from fibroin, expanding its applicability in drug delivery. Partial hydrolysis, such as alkaline treatment, produces shorter fibroin chains with improved enzyme-inhibitory activities (e.g., tyrosinase inhibition), metal-chelating abilities, and better moisture retention [237]. Its rich amino acid profile permits extensive chemical modifications, including attaching biofunctional groups or crosslinking with other polymers to customize biological and mechanical properties [274,275,276]. Additionally, fibroin’s high structural stability allows it to be blended with other hydrogels, enhancing mechanical strength and durability [277].

## 5. 3D Printing—A Gateway to Innovation in Wound Treatment

### 5.1. 3D Printing for Personalization and Structural Control

**Figure 4:**
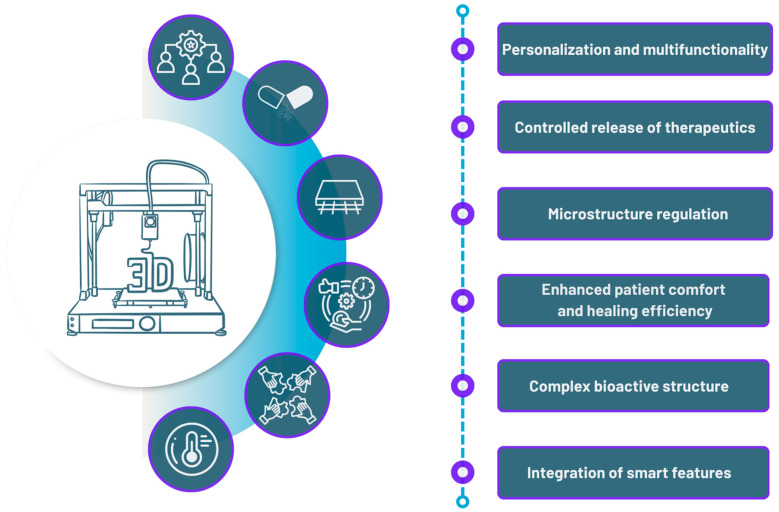
Advantages of 3D-Printed Wound Dressings.

3D printing offers numerous advantages for wound dressings (Figure 4). A high level of control over both the micro- and macrostructures of wound dressings is a significant asset over alternative methods that provide less precise structural control [278]. Wounds are often irregularly shaped, and traditional dressings typically require manual trimming to match the wound’s contour, usually only in two dimensions [279]. In contrast, 3D printing allows for the creation of hydrogel structures tailored to the wound’s topography in three dimensions, ensuring a more accurate and functional fit. A significant benefit of 3D printing is its precise control over microstructure [278]. For instance, while electrospinning permits some regulation of fiber diameter and porosity through process parameters, the resulting fiber orientation is generally randomized or collateral, and controlling fiber composition over those two orders is challenging [279]. Freeze-drying, on the other hand, does not ensure uniform pore distribution and offers limited control over pore architecture [280,281]. Managing microstructure is crucial for replicating the natural architecture of tissue, thereby supporting more effective tissue regeneration. Additionally, improved structural precision can enhance the mechanical properties of the hydrogel, which are attributes that depend not only on the polymer used but also on the internal architecture of the dressing [282,283].

From a personalization perspective, not only the shape but also the ability to tailor the dressing in terms of polymer composition and APIs is a significant advantage of 3D printing. APIs can be incorporated into a single dressing, where different substances can be either spatially separated, delivered in a balanced manner, or released immediately in a high dose. Such capabilities are enabled by selecting an appropriate polymer or by adjusting the degree of matrix crosslinking [24,284,285,286].

One key benefit of 3D printing technology is its ability to go beyond large-scale manufacturing. Adding 3D printers to wound care centers allows for on-site creation of custom dressings right before use, which is vital for personalized treatment. Their compact size and simple operation, automated mainly by computer programs, make them easy to integrate into clinical settings [287]. Consequently, wound dressings can be precisely tailored to fit the patient’s specific wound shape and medical condition, while also allowing real-time modifications as the wound changes. This prompt response significantly enhances treatment success and patient comfort [288]. Additionally, point-of-care 3D printing makes it possible to embed living cells into bioinks, which need to be used soon after printing [289]. Finally, local manufacturing helps reduce logistical issues such as distribution and storage costs, which are often associated with traditional production methods [290].

### 5.2. Stimulus-Responsive Hydrogels and 4D Printing

Printed hydrogels can facilitate more intricate release profiles of loaded APIs by enabling the construction of matrices built from various materials responsive to specific stimuli. Some hydrogel dressings respond to near-infrared (NIR) irradiation, which triggers the release of drugs by converting absorbed light into heat. This thermal energy promotes matrix degradation and enhances molecular diffusion [291]. Hydrogel systems that are temperature-sensitive, undergoing a sharp sol–gel transition in response to either body heat or externally applied heat [292]. Adding magnetic materials, such as iron oxides, transition metal alloys, or ferrites, can make hydrogels responsive to magnetic fields. These magnetic hydrogels can be directed to target sites, potentially minimizing off-target side effects. Magnetic stimulation can also regulate drug release by causing relaxation of polymer chains or inducing mechanical vibrations within the hydrogel. These deformations are controllable through switching magnetic fields on and off, allowing pulsatile or sustained drug delivery [293,294]. Additionally, exposure to high-frequency alternating magnetic fields can generate localized heating, which further aids drug release, similar to NIR irradiation [295,296]. Electroresponsive hydrogels enable precise control over drug delivery, with the amount of released API often linked to the applied voltage [297,298,299,300]. Although these systems need external stimulation, some hydrogel types can also react to internal wound cues. pH-responsive hydrogels are particularly promising in wound care. The pH of the wound microenvironment fluctuates depending on the stage of healing and the presence of infection. Infected wounds tend to have an alkaline pH, whereas healing wounds, particularly in later stages, exhibit more acidic conditions similar to healthy skin [301,302]. Hydrogels made from pH-sensitive polymers can be designed to release drugs in response to these environmental shifts. Polymers with acidic groups swell at pH levels above their pKa, while basic polymers swell below their pKa. This swelling depends on the ionization state of functional groups—deprotonation in acids and protonation in amines, which affects drug release rates [292,303].

The properties mentioned above, which describe certain hydrogel systems, all with the concept of 4D printing—an evolution of traditional 3D printing. While 3D printing allows for creating static, three-dimensional structures tailored to specific topographies, 4D printing adds a dynamic, stimulus-responsive feature. By integrating environmental or external triggers into the design, these smart hydrogels can modulate the release of APIs not only in response to time but also in reaction to specific physiological or physical signals. This innovation advances the goal of personalized and adaptive wound care toward practical implementation [292].

### 5.3. Biosensing Integration in Printed Dressings

An innovative aspect of 3D-printed dressings is the integration of structural benefits with biosensing functions, enabling real-time monitoring of wound healing. Different advanced materials are designed to detect pH changes in wounds by showing color shifts in embedded indicator dyes or by measuring the swelling of pH-sensitive hydrogel matrices [304,305]. Due to the significantly impaired wound healing process in patients with type 2 diabetes, there are ongoing ideas to use 3D-printed dressings for monitoring glucose levels directly within the wound environment [306]. Creating multilayered structures allows for integrating various sensing functions like temperature measurement, swelling detection, and glucose monitoring, within a single hydrogel-based system [307]. Integrating biosensing with structural features transforms traditional wound dressings into active, interactive platforms. These intelligent systems not only support healing and regulate moisture but also enable early detection of problems, such as infections or inflammation [308,309]. By continuously monitoring physiological signals, such as pH, glucose levels, or temperature, clinicians can provide valuable data for personalized treatment and make real-time decisions. Additionally, with advancements in wireless communication and data transfer, future 3D-printed hydrogel dressings could enable remote wound monitoring, offering significant benefits for managing chronic wounds and patients with limited access to frequent clinical care [310,311].

The innovations enabled by 3D printing are not just theoretical ideas but are supported by ongoing experimental work. To provide a visual summary of these methods, Table 5 shows selected examples from experimental studies that demonstrate 3D-printed hydrogel dressings.

## 6. Limitations

The major limitation lies in maintaining the stability of the amorphous form. Ensuring thermodynamic and kinetic stability is crucial both during ASD preparation and after incorporation into the hydrophilic environment of a hydrogel [318]. In aqueous settings, polymers can undergo hydrolysis or absorb moisture, leading to recrystallization and loss of solubility benefits. This requires the careful selection of moisture-resistant polymers or the design of anhydrous systems that are activated immediately before use. The polymer matrix and hydrogel structure greatly influence whether drug release is immediate or sustained. Achieving a release profile suited to the API’s therapeutic window demands careful adjustment of polymer composition and hydrogel architecture [319,320]. Both matrix degradation and diffusion mechanisms must be considered. Formulation must balance fluidity for processing (e.g., 3D printing) with post-application stability. Rheological properties need to support both structural integrity and reliable, controlled release throughout therapy [31]. Hydrogel compositions with high water content are at risk for microbial growth. To obtain a sterile final product, aseptic manufacturing or additional sterilization is necessary, along with antimicrobial strategies to prevent biocontamination. While natural polymers like gelatin or collagen provide bioactive benefits, they can be enzymatically degraded by microbial or host enzymes. This degradation may undesirably accelerate drug release or weaken structural stability in vivo [321]. Complex manufacturing processes that combine ASD production, hydrogel formulation, and sterile final product fabrication may pose scale-up challenges, including cost, reproducibility, and regulatory compliance. Hydrogel-based wound dressings with ASDs may be classified as combination products, especially when they exhibit both a pharmacological effect (via the API) and a physical function (such as moisture retention or tissue support). This dual nature can complicate approval pathways by involving multiple regulatory categories. Additionally, maintaining consistency in key quality attributes, such as drug content uniformity, sterility, mechanical stability, and release kinetics, is vital, particularly when employing advanced manufacturing techniques like 3D printing. Patient-specific factors, such as exudate volume, infection presence, pH, or tissue ischemia, can unpredictably affect hydrogel integrity, API release, and overall therapeutic outcomes. Optimal designs may require diagnostic sensing or adaptive formulations to respond dynamically to the local wound environment [322].

## 7. Conclusions

Amorphous dispersions expand the therapeutic potential of poorly soluble compounds in wound care, especially when incorporated into hydrogel matrices. This combination improves local drug availability and helps maintain effective concentrations within the wound microenvironment. Challenges include ensuring stability, creating uniform ASD—hydrogel formulations, and meeting manufacturing and microbiological quality standards.

Future progress depends on translating promising in vitro results into solid in vivo evidence, followed by clinical validation and regulatory approval. Advances in hydrogel engineering and 3D printing offer additional opportunities for personalization, but their clinical use requires a structured assessment of safety, efficacy, and scalability. If these steps are achieved, ASD-integrated hydrogels could accelerate wound healing, reduce complications, and promote more personalized treatment approaches.

## Figures and Tables

**Table 1:** Key pathomechanisms in chronic wounds.

Mechanism	Clinical Relevance	Sources
Bacterial infections	Persistent microbial presence delays healing and increases the risk of complications.	[43,44]
Biofilm formation	Biofilms protect bacteria, sustain inflammation, and reduce the effectiveness of antibiotics, a significant factor in chronic infections.	[45,46,47]
Debridement	Removes necrosis and biofilm, resets the wound bed, and enhances response to therapies.	[43,48,49]
Inflammation	Dysregulated, prolonged inflammation keeps wounds in a non-healing state.	[50,51]
Oxidative stress	Excess ROS damages tissue, sustains inflammation, and hinders healing.	[52,53,54]
Hypoxia	Chronic hypoxia impairs cellular function, including fibroblasts; HIF-1α guides adaptive repair.	[55,56]
Angiogenesis	New vessel growth restores blood flow and promotes granulation and epithelialization.	[57,58]
Wound closure	Complete closure is the most significant patient-centered outcome; it supports regulatory and clinical decisions.	[59,60]
Melanin overproduction	Post inflammatory hyperpigmentation is linked to injury and inflammation; it impacts quality of life and clinical evaluation of wounds.	[61,62]

**Table 2:** Published evidence on solubility enhancement through amorphization methods.

API	Polymer	Method of Amorphization	Improvement in Solubility	Metric Type	Source
α-Lipoic acid	Soluplus^®^	Lyophilization	12.7 ± 5.8% → 87.7 ± 5.5%	Percentage	[145]
Pterostilbene	PVP K30	Ball milling	~1417-fold (vs. native)	Fold Increase	[146]
Chrysin	Plasdone^®^ S630	Solvent evaporation	20–25% → 60–80% (pH 6.8)	Percentage	[147]
Magnolol	HPMC-AS	Antisolvent coprecipitation	C_max_ 1.76×; AUC_0–48_ 2.17×	C_max_ and AUC	[148]
Paracetamol	PVP-VA	Spray drying	Up to 6× vs. saturated solution	Fold Increase	[149]
Ciprofloxacin	PVP	Electrospinning	41 ± 3% → 94 ± 6% (12× vs. raw ciprofloxacin)	Percentage and Fold Increase	[150]
Curcumin	Soluplus^®^	Hot Melt Extrusion	Up to 9× vs. pure curcumin	Fold Increase	[151]
Spironolactone, nifedipine	Ethyl cellulose	Electrospinning	75 mg/L vs. 5.9 and 22 mg/L	Absolute Concentration	[152]
Rafoxanide	PVP K25	Spray drying	Physical mixture: 0%; ASD: ~100%	Percentage	[153]
Cannabidiol	Eudragit^®^ EPO	Hot Melt Extrusion	80% (35× vs. pure CBD)	Percentage and Fold Increase	[154]

PVP—Polyvinylpyrrolidone; HPMC-AS—Hydroxypropyl methylcellulose–succinic acid; PVP-VA—Polyvinylpyrrolidone–vinyl acetate.

**Table 3:** Representative Techniques for the Preparation of Amorphous Solid Dispersions.

Method	Temperature	Solvent	Advantages	Limitations	Sources
Hot Melt Extrusion	Moderate/ High	No	Solvent-free and scalable; suitable for APIs prone to oxidation and hydrolysis; no need for further processing; high product purity	Not suitable for thermolabile APIs; high energy consumption; requires high flow properties of raw materials; a large batch size is needed for analysis	[159,160,161,162,163]
Spray Drying	Moderate	Yes (mostly organic)	High surface area, fast, and effective for industrial scale-up	Use of organic solvents; risk of partial crystallization; requires careful condition optimization	[164,165,166,167,168]
Freeze Drying	Low	Yes (mostly aqueous)	Suitable for thermolabile APIs; enables creation of porous structures	Slow; high energy consumption; mostly suitable for water-soluble substances	[169,170,171,172]
Electrospinning	Low	Yes (mostly organic)	Allows nanostructure formation; suitable for thermolabile APIs	Complex setup, slow process, difficult to scale up	[173,174,175,176]
Supercritical Fluid Technology	Variable	No	Leaves no solvent residues; suitable for oxidation- and hydrolysis-sensitive APIs	High cost; limited infrastructure availability	[177,178,179,180,181]
KinetiSol^®^	Medium/ High	No	Short exposure to elevated temperatures, rapid amorphization; suitable for APIs with high melting points or poor thermal stability	Requires specialized equipment; potential degradation of heat- or shear-sensitive APIs; risk of local overheating for materials with low thermal conductivity	[182,183,184,185]
Ball milling	Low/ Moderate	No	Does not require additional excipients; easy to use; applicable at lab scale	Possible temperature rise during milling; limited amorphization efficiency; hard to scale up; relatively high recrystallization risk	[186,187,188,189,190]

**Table 4:** Functional characteristics of natural and synthetic polymers in wound management.

Polymer	Properties	Impact on Wound Healing as a Dressing	Sources
PVP	Adhesive to skin; prevents recrystallization; chemically and biologically inert	Absorbs exudates; facilitates removal of necrotic tissues	[211,212,213]
PVA	Adhesive to skin; chemically and biologically inert; biodegradable	Absorbs exudates; facilitates removal of necrotic tissues	[213,214]
PAA	Chemically and biologically inert; biodegradable; pH-responsive; mucoadhesive	Absorbs exudates; facilitates removal of necrotic tissues	[200,215,216]
Collagen	Biocompatible; degradable in wound environment; natural component of skin; high similarity to natural ECM; hygroscopic properties; substrate for endogenous MMPs	Supports cell migration, proliferation, and differentiation; facilitates blood clot formation and immune response; promotes M2 macrophage, angiogenesis (type I), and epithelialization; reduces ECM degradation and sustains matrix remodeling; absorbs exudates; promotes removal of necrotic tissues	[208,217,218,219]
Gelatin	Biocompatible; degradable in wound environment; rheological and thermal stability in pH range 5–9	Supports cell migration and proliferation; facilitates blood clot formation; absorbs exudates; promotes removal of necrotic tissues	[220,221,222,223]
Chitosan	Biocompatible; hygroscopic; biodegradable; nontoxic; thermally stable; soluble in acidic solutions; mucoadhesive; hemostatic	Supports cell migration, proliferation, and differentiation; facilitates blood clot formation; provides antibacterial and antiinflammatory effects; absorbs exudates; promotes removal of necrotic tissues	[224,225,226,227,228,229]
Sodium alginate	Biocompatible; swellable; biodegradable; nontoxic; pH-sensitive; gelates in acidic conditions; ion-crosslinked forms may release ions at wound site	Absorbs large amounts of exudate; keeps wound moist; reduces oxidative stress	[230,231,232,233]
Silk fibroin	Biocompatible; biodegradable; nontoxic; inhibits tyrosinase; chelates ferrous ions; hemostatic; improves hydrogel mechanical strength	Supports cell migration and proliferation; facilitates blood clot formation; exhibits anti-inflammatory activity; promotes ECM formation, angiogenesis, collagen synthesis, re-epithelialization, and wound contraction	[234,235,236,237,238,239,240,241]

PVP—polyvinylpyrrolidone; PVA—polyvinyl alcohol; PAA—polyacrylic acid; ECM—extracellular matrix; MMPs—metalloproteinases.

**Table 5:** Examples of 3D-Printed Hydrogel Systems with Functional Characteristics Relevant to Wound Healing.

Polymeric Compound	Picture	Comment	Source
Gelatin methacryloyl and dialdehyde-functionalized polyurethane	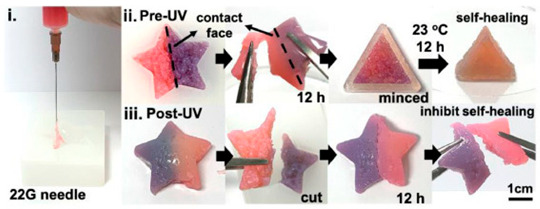	Example of a self-healing hydrogel, where fragments fuse upon contact over time, allowing the formation of more complex three-dimensional structures. Exposure to UV radiation stabilizes the structure but also removes the ability for further self-repair.	Reproduced with permission from [312]
(a,b) Sodium alginate; (c,d) Sodium alginate and active ingredients	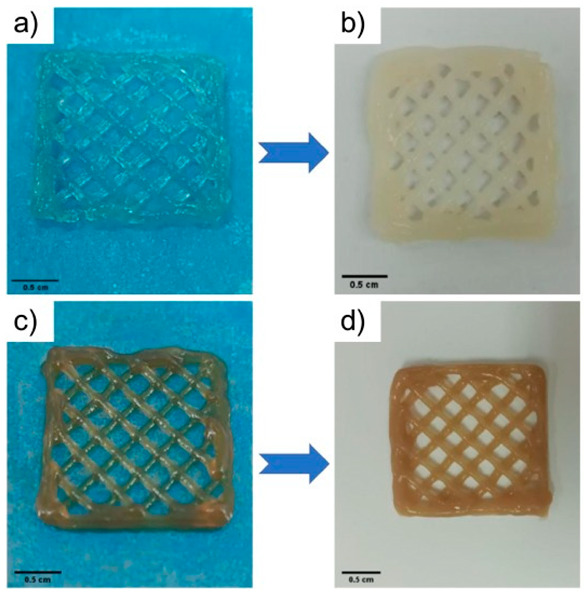	Photographs show changes in printed dressings for (a) and (c) after crosslinking with calcium chloride by immersing the structures in a crosslinking solution in the image (b) and (d). This process enhances mechanical strength and may also influence the release of active substances as well as the degradation of the hydrogel.	Reproduced with permission from [313]
Acrylamide and chitosan modified with methacryloyl groups	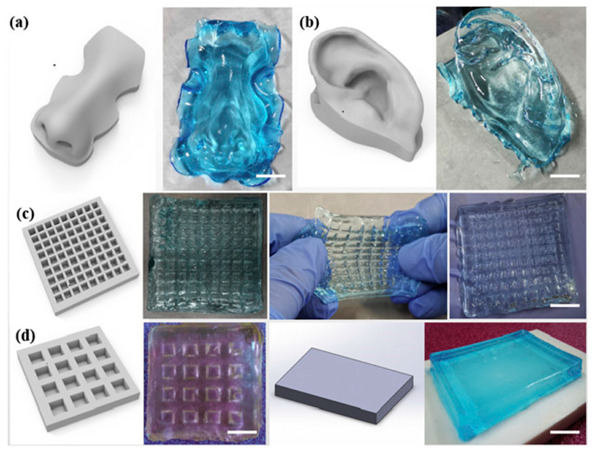	Structures produced using 3D printing with digital light processing allow the fabrication of high-resolution, complex spatial architectures. The applied polymers and crosslinking strategy impart shape-memory properties, enabling the structures to return to their original form after deformation. (a) the Computer Aided Design (CAD) model and the printed construct of nose. (b) the CAD model and the printed construct of ear auricle with helical fold. (c) and (d) Lattice structures of chitosan modified with methacryloyl groups/polyacrylamide hydrogels with different mesh size produced by digital light processing.	Reproduced with permission from [314]
Chitosan and pectin	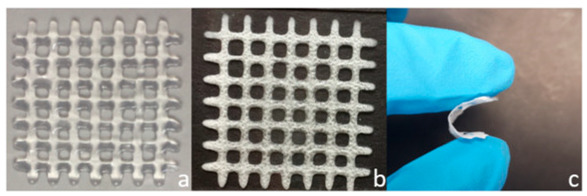	3D-printed dressings, after shaping, can undergo lyophilization. Water removal ensures the stability of the intended shape, results in a more compact size, and decreases the risk of hydrolysis of polymers and active substances, as well as microbial growth. The lyophilized dressing can be rehydrated before use or regenerated by wound exudate at the application site. (a) freshly printed, (b) lyophilised and (c) flexibility of a lyophilised scaffold.	Reproduced with permission from [315]
Poly(N-isopropylacrylamide), precursors, sodium alginate and methylcellulose	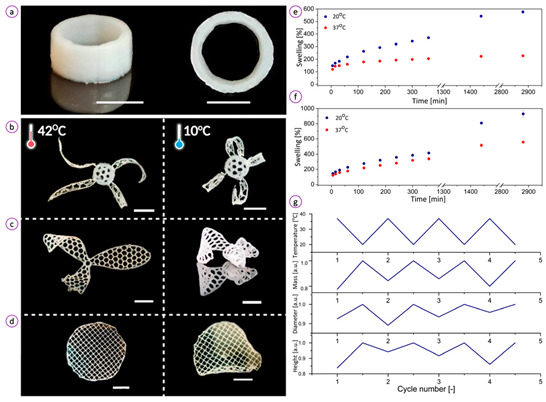	An example of thermoresponsive hydrogels that alter their shape and size with temperature changes. Printed objects can be programmed to show different actuation behaviors at various temperatures (42 °C and 10 °C). Cyclic swelling happens at 20 °C and deswelling occurs at 37 °C. (a) 3D-printed thermoresponsive tube showing high ink printability; diameter changes with temperature. (b) Flower-like object with thermoresponsive petals and inert core. (c) Hydrogel propeller. (d) Hydrogel disc. Objects (b–d) exhibit programmable actuation at 42 °C and 10 °C. Scale bars: 1 cm. (e, f) Swelling rates at 20 °C and 37 °C in water and PBS. (g) Cyclic swelling (20 °C) and de-swelling (37 °C) measured as changes in height, diameter, and mass.	Reproduced from Nizioł et al., under CC BY license [316]
Gelatin	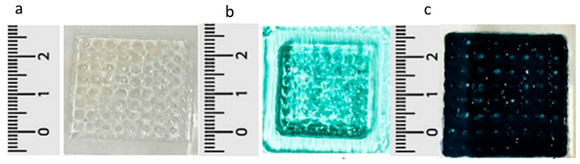	Example of polymeric hydrogel crosslinking, where the degree of crosslinking correlates with color intensity, which is proportional to the polymer concentration. Crosslinking agent: genipin. (a) Non-crosslinked gelatin hydrogel; (b) crosslinked 8% gelatin hydrogel; (c) crosslinked 10% gelatin hydrogel. Crosslinked with 0.3% genipin.	Reproduced from Taghdi et al., under CC BY license [317]
Sodium alginate	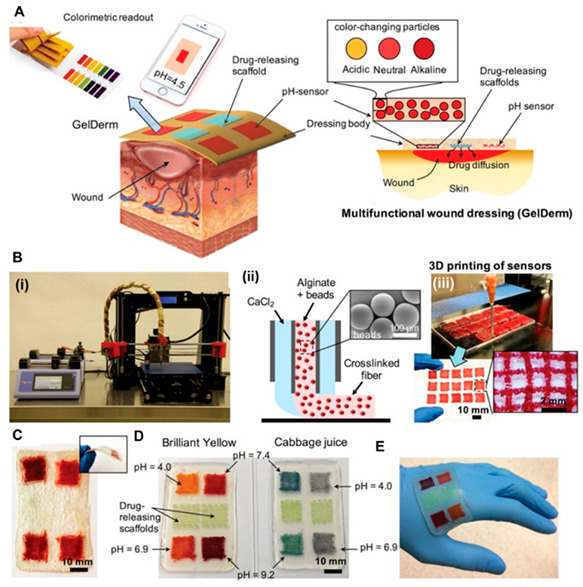	Illustration of a concept where chemical pH indicators help monitor wounds in situ while also delivering antibacterial agents. This method is connected to portable devices for real-time color-change analysis. (d) Synthetic Brilliant Yellow and naturally derived cabbage juice served as model pH indicators for creating the sensors. Sensor arrays enable spatial detection of pH variations at the wound site. Drug-eluting scaffolds release high doses of antibiotics locally to eliminate residual bacteria each time the dressing is changed. (A) Schematic of GelDerm treatment with pH-sensitive and drug-eluting components. (B-i–iii) Porous sensors fabricated via 3D bioprinter with co-axial nozzle; arrays allow large-scale dressing production. (C) Dressings can be lyophilized and sterilized for storage. (D) Brilliant Yellow and cabbage juice used as pH indicators; sensor arrays detect spatial pH variations, while drug-eluting scaffolds release antibiotics to eradicate residual bacteria. (E) GelDerm maintains conformal contact with irregular surfaces.	Reproduced from Malekmohammadi et al., under CC BY license [304]

## Data Availability

Not applicable.

## Abbreviations

The following abbreviations are used in this manuscript:

ASDs	Amorphous Solid Dispersions
3D	3-Dimensional
API	Active Pharmaceutical Ingredient
HIF-1α	Hypoxia-Inducible Factor 1 α
ROS	Reactive Oxygen Species
EPS	Extracellular Polymeric Substance
M1	Proinflammatory Macrophages
TNF-α	Tumor Necrosis Factor α
ILs	Interleukines
MMPs	Matrix Metalloproteinases
ECM	Extracellular Matrix
VEGF	Vascular Endothelial Growth Factor
SDF-1	Stromal Cell-derived Factor-1
Ang-1	Angiopoietin-1
Ang-2	Angiopoietin-2
TOT	Topical Oxygen Therapy
HBOT	Hyperbaric Oxygen Therapy
THBS-1	Thrombospondin-1
TRP-1	Tyrosinase-Related Protein-1
TRP-2	Tyrosinase-Related Protein-2
FDA	Food and Drug Administration
PVP	Polyvinylpyrrolidone
HPMC-AS	Hydroxypropyl methylcellulose–succinic acid
PVP-VA	Polyvinylpyrrolidone–vinyl acetate
EC	Ethyl cellulose
HME	Hot Melt Extrusion
SDF	Supercritical Fluid Technology
BSE	Bovine Spongiform Encephalopathy
PVA	Polyvinyl Alcohol
PAA	Polyacrylic Acid
NIR	Near-Infrared
CAD	Computer Aided Design
